# Psychological distress and its relationship with non-adherence to TB treatment: a multicentre study

**DOI:** 10.1186/s12879-015-0964-2

**Published:** 2015-07-01

**Authors:** Grant Theron, Jonny Peter, Lynn Zijenah, Duncan Chanda, Chacha Mangu, Petra Clowes, Andrea Rachow, Maia Lesosky, Michael Hoelscher, Alex Pym, Peter Mwaba, Peter Mason, Pamela Naidoo, Anil Pooran, Hojoon Sohn, Madhukar Pai, Dan J. Stein, Keertan Dheda

**Affiliations:** Lung Infection and Immunity Unit, Division of Pulmonology & UCT Lung Institute, Department of Medicine, University of Cape Town, H47 Old Main Building, Groote Schuur Hospital, Observatory, Cape Town, 7925 South Africa; Department of Immunology, University of Zimbabwe College of Health Sciences, Harare, Zimbabwe; University Teaching Hospital, Lusaka, Zambia; Institute for Medical Research & Training, Lusaka, Zambia; National Institute of Medical Research, Mbeya Medical Research Centre, Mbeya, Tanzania; Division of Infectious Diseases and Tropical Medicine, Medical Centre of the University of Munich (LMU), Munich, Germany; Department of Medicine, University of Cape Town, Cape Town, South Africa; German Centre for Infection Research (DZIF), Munich, Germany; South African Medical Research Council, Durban, South Africa; KwaZulu Research Institute for Tuberculosis and HIV (K-RITH), Durban, South Africa; Biomedical Research & Training Institute, Harare, Zimbabwe; Population Health, Health Systems and Innovation (PHHSI)/HIV/STIs and TB (HAST) Research Programmes, Human Sciences Research Council, Cape Town, South Africa; McGill International TB Centre & Department of Epidemiology & Biostatistics, McGill University, Montreal, Canada; Department of Psychiatry & Mental Health, University of Cape Town, Cape Town, South Africa

**Keywords:** Tuberculosis, Psychological distress, Socioeconomic status, Treatment non-adherence

## Abstract

**Background:**

The successful cure of tuberculosis (TB) is dependent on adherence to treatment. Various factors influence adherence, however, few are easily modifiable. There are limited data regarding correlates of psychological distress and their association with non-adherence to anti-TB treatment.

**Methods:**

In a trial of a new TB test, we measured psychological distress (K-10 score), TB-related health literacy, and morbidity (TBscore), prior to diagnosis in 1502 patients with symptoms of pulmonary TB recruited from clinics in Cape Town (*n* = 419), Harare (*n* = 400), Lusaka (*n* = 400), Durban (*n* = 200), and Mbeya (*n* = 83). Socioeconomic, demographic, and alcohol usage-related data were captured. Patients initiated on treatment had their DOTS cards reviewed at two-and six-months.

**Results:**

22 %(95 % CI: 20 %, 25 %) of patients had severe psychological distress (K-10 ≥ 30). In a multivariable linear regression model, increased K-10 score was independently associated with previous TB [estimate (95 % CI) 0.98(0.09-1.87); *p* = 0.0304], increased TBscore [1(0.80, 1.20); *p* <0.0001], and heavy alcohol use [3.08(1.26, 4.91); *p* = 0.0010], whereas male gender was protective [-1.47(−2.28, −0.62); *p* = 0.0007]. 26 % (95 % CI: 21 %, 32 %) of 261 patients with culture-confirmed TB were non-adherent. In a multivariable logistic regression model for non-adherence, reduced TBscore [OR (95 % CI) 0.639 (0.497, 0.797); *p* = 0.0001], health literacy score [0.798(0.696, 0.906); *p* = 0.0008], and increased K-10 [1.082(1.033, 1.137); *p* = 0.0012], and heavy alcohol usage [14.83(2.083, 122.9); *p* = 0.0002], were independently associated. Culture-positive patients with a K-10 score ≥ 30 were more-likely to be non-adherent (OR = 2.290(1.033-5.126); *p* = 0.0416].

**Conclusion:**

Severe psychological distress is frequent amongst TB patients in Southern Africa. Targeted interventions to alleviate psychological distress, alcohol use, and improve health literacy in newly-diagnosed TB patients could reduce non-adherence to treatment.

**Electronic supplementary material:**

The online version of this article (doi:10.1186/s12879-015-0964-2) contains supplementary material, which is available to authorized users.

## Background

Tuberculosis (TB) is a preventable and curable disease, yet it is responsible for over 1.3 million deaths every year [1]. Patients with TB are treated for six to nine months with antibiotics. Although heavy alcohol use and smoking are independently associated with an increased risk of TB infection [2–7], screening, care and counselling for these conditions, as well as care for psychiatric illness, is poorly integrated with TB care, and is infrequently available to patients attending clinics in high burden, resource-limited settings.

Adherence to anti-TB treatment can widely vary, with some studies reporting rates of default of up to 50 % [8]. A broad range of patient-specific demographic, financial, and behavioural factors, as well setting-and regimen-specific factors are known to influence adherence [9, 10], however, most of these, such as household income, are not easily alterable by healthcare providers. Furthermore, TB patients are known to consider defaulting several times over the course of treatment, with the intensity of their motivation to complete their regimen fluctuating [11, 12]. Those who default are, compared to patients who are adherent, at increased risk of morbidity and mortality, are more likely to develop drug resistance, and are more likely to transmit TB.

A high prevalence of psychological distress (including symptoms depression and anxiety) has been documented amongst TB patients. One South African study found 60 % of patients to have symptoms of depression [13]. Another found 33 % of patients to have symptoms of severe psychological distress [14], and another demonstrated a trend of increased adverse life events associated with increased TB incidence [15]. The Kessler K-10 questionnaire, which has been validated in a variety of settings as part of population-level mental health surveys [16], is a tool for population-level screening of people who are likely to meet formal DSM-IV definitions for anxiety or depressive disorders, and those who have sub-clinical psychiatric illness [17]; yet the K-10 questionnaire has not been widely used to study the interaction between mental health and infectious diseases.

Psychological distress, which is known to down-regulate the immune response [18], may, in addition to making patients less likely to seek care, influence anti-TB treatment adherence and clinical outcome (e.g., culture-conversion or death) [19]. Additionally, TB patients who are psychologically distressed might congregate in settings where transmission is more likely to occur, such as homeless shelters or informal pubs or bars, and hence might be more likely to be infected with TB. There is, however, little known about correlates of psychological distress, including socioeconomic factors, in the context of TB. Finally, the linkage between psychological distress and adverse clinical events, such as treatment non-adherence, is poorly studied.

We did a large, five-site clinical study that examined the effect of a new TB test in patients seeking care for TB in primary care settings in five sites in Southern Africa [20]. Here we report on psychological distress in this cohort. We primarily hypothesised that patients who had higher levels of psychological distress would be more likely to be non-adherent to their anti-TB treatment. We also explored the association between psychological distress, clinical characteristics (such as TB-related morbidity), socioeconomic characterstics (such as income, educational level, health literacy, and unemployment), and healthcare seeking behaviour, such as the duration of symptoms that passed before patients sought care.

## Methods

### Study design

We conducted a pragmatic, randomised (1:1), parallel-arm, multi-centric trial between April 2011 and October 2012, during which patients received either Xpert MTB/RIF [21], a new World Health Organisation-approved test, or sputum smear microscopy for the frontline diagnosis of TB [20]. The trial was registered on Clinicaltrials.gov (identifier NCT01554384).

We collected clinical, psychosocial, and socioeconomic information at recruitment, and after two months and six months of anti-TB treatment.

#### Study sites and inclusion criteria

After written informed consent, we consecutively enrolled patients ≥18 years who presented to periurban primary-care TB clinics in Cape Town (South Africa), Durban (South Africa), Harare (Zimbabwe), Lusaka (Zambia), and Mbeya (Tanzania). The study was approved by local ethics committees at each site. We enrolled consenting patients who had symptom(s) of pulmonary TB according to predefined WHO criteria [22, 23], who could expectorate at least two sputum specimens, and who had not been on anti-TB treatment within the last 60 days.

### Procedures

Patients were offered voluntary testing and counselling for HIV at recruitment. All patients received a package of diagnostic tests for TB (chest radiography, liquid culture, and microscopy or Xpert MTB/RIF). If a positive bacteriological result was obtained, the patient was referred for the initiation of anti-TB treatment. Patients who were not bacteriological test-positive for TB were referred for routine clinical review, and could still be initiated on treatment based on clinical signs and symptoms at their doctor’s discretion.

#### Adherence to anti-TB treatment

Nurses at each site reviewed the patients DOTS clinic card at two month and six months after the initiation of anti-TB treatment. Patients who were noted to have missed at a scheduled DOTS visit were classified as non-adherent.

#### Collection of psychosocial and economic data

All patients had their demographic and clinic information captured using a validated case record form. The Kessler K-10 questionnaire [24, 25], which measure psychological distress within the last 30 days (Additional file 1: Table S1) was, together with a standardised TB health literacy questionnaire (Additional file 1: Table S2) [26], administered by nurses in English or the patient’s mother tongue. TB-related morbidity was measured using the validated TBscore symptom scoring system [27, 28] (Additional file 1: Table S3). Information about cigarette and alcohol consumption, educational level, personal- and household-income were also captured. Patients started on anti-TB treatment were followed-up at two- and six-month post-enrolment by study staff, at which time their TB-related morbidity was measured.

#### Case definitions

Patients were classified as definite TB if sputum collected at recruitment grew acid-fast bacilli in liquid culture (Mycobacteria Growth Indicator Tube, MGIT; BD Microbiology Systems, USA), which was identified as *Mycobacterium tuberculosis* complex [29].

#### Statistical analyses

Fisher’s exact test with mid-P correction was used for comparisons between proportions. We developed a series of multivariable regression models to examine independent associates of: (i) psychological distress (K-10 score); (ii) non-adherence to anti-TB treatment; (iii) improvement in morbidity after six months of treatment; (iv) mortality; (v) cough duration prior to seeking care; and (vi) whether patients reported at follow-up their employment had been affected by their TB. We adjusted for potential confounding, intra-site interactions, and clustering using fixed effects. Analyses were performed using OpenEpi (version 2.3.1) [30], Graphpad Prism (version 6.0; GraphPad Software, USA), and R (version 3.0) [31]. All statistical tests are two-sided at α = 0.05.

#### Role of the funding source

The European and Developing Countries Clinical Trials Partnership had no role in study design, data collection, data analysis, data interpretation, or writing of the report. The corresponding author had full access to all the data in the study and had final responsibility for the decision to submit for publication.

## Results

### Demographic, educational, and economic characteristics

**Fig. 1:**
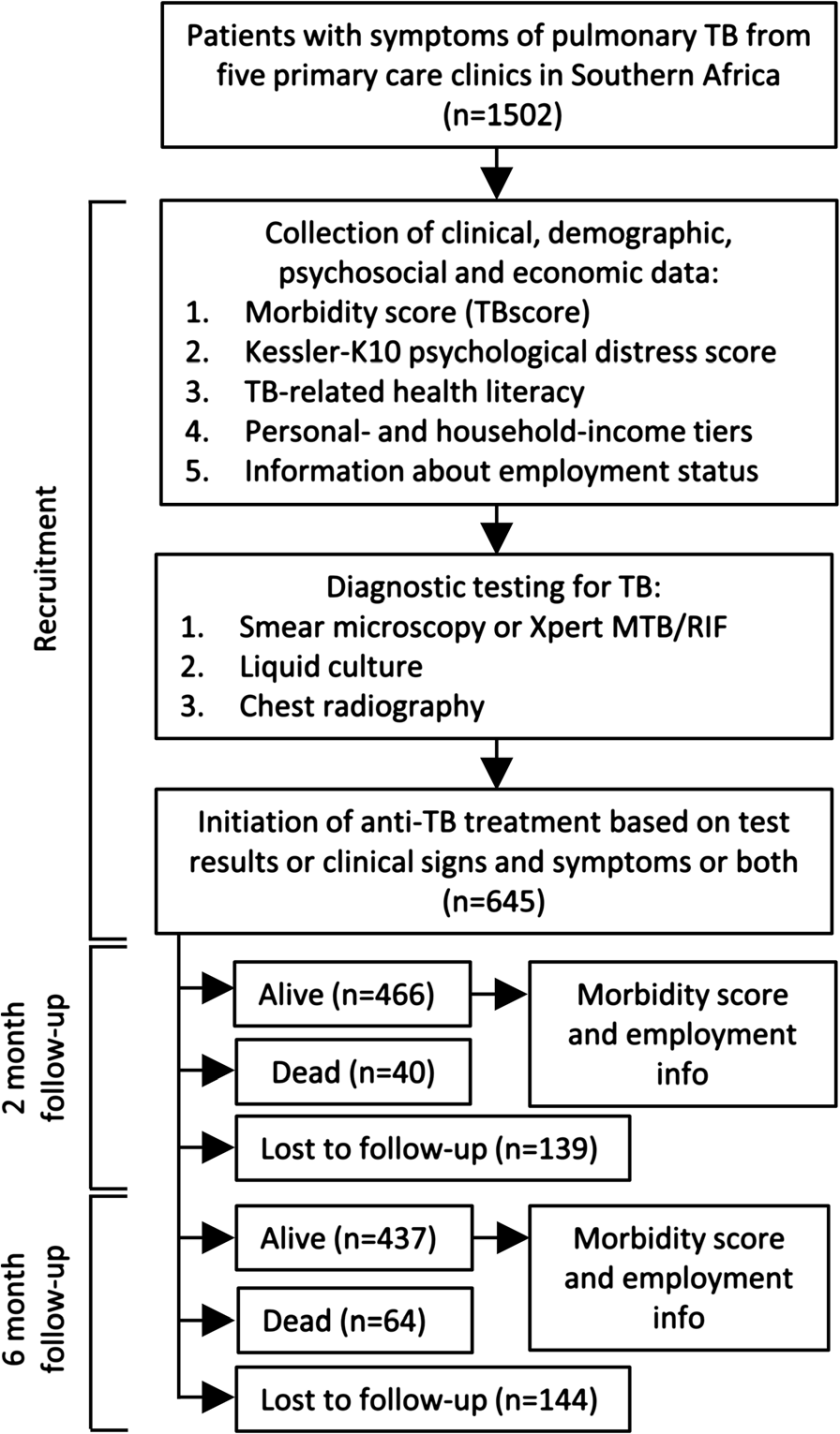
Study profile

A patient flow diagram is shown in Fig. 1. We enrolled 1502 patients; the demographic, clinical, and socioeconomic characteristics of which are summarised in Table 1. Most patients were men (57 %), living with HIV (60 %), and had TB for the first time (74 %). Twenty eight percent of patients self-reported to be tobacco smokers, and 48 % said that they did not consume alcohol. Most patients had attained a middle school (28 %) or high school qualification (26 %), and had a median [interquartile range (IQR)] TB health literacy score of 6 (4–8) out of 13. Most patients were unemployed (55 %), reported a personal income falling within the lowest tier (43 %; <600 ZAR per month for the South African sites or <100 US$ per month for the others sites), and a household income falling within the second lowest tier (39 %; 600–3000 ZAR per month for the South African sites or 100–300 US$ per month for the other sites). Twenty four percent of patients had culture-confirmed TB.

**Table 1:** Cohort demographic, clinical, psychosocial and economic characteristics at baseline by site

	Gugulethu TB clinic (Cape Town, South Africa)	Mabvuku polyclinc (Harare, Zimbabwe)	Kanyama TB clinic (Lusaka, Zambia)	St. Mary’s day clinic (Durban, South Africa)	Ifisi day clinic (Mbeya, Tanzania)	Overall
Number of patients	419	400	400	200	83	1502
**Demographic and clinical information**						
Age [median (IQR)]	39 (31–49)	38 (32–45)	35 (30–41)	37 (30–50)	37 (31–54)	37 (30–46)
Male (%)	259 (62)	185 (46)	269 (67)	104 (52)	42 (51)	859 (57)
Previously had TB (%)	178 (43)	67 (17)	85 (21)	52 (26)	2 (1)	384 (26)
HIV-infected^a^ (%)	133 (32)	324 (81)	268 (67)	121 (61)	49 (59)	895 (60)
TB symptom score [TBscore; median (IQR)]	4 (3–5)	4 (3–5)	5 (4–7)	5 (4–6)	7 (4–9)	
Culture-confirmed TB cases (%)	74 (18)	77 (19)	152 (38)	35 (18)	29 (35)	367 (24)
Kessler K-10 score						
Median (IQR)^e^	20 (16–24)	30 (25–35)	16 (10–24)	18 (15–22)	29 (23–33)	22 (16–29)
Graded as severe (≥30) (%)	31/413 (8)	201/400 (50)	49/399 (12)	16/199 (8)	38/74 (51)	335 (22)
**Substance use**						
Tobacco smoker (%)	249 (60)	32 (8)	92 (23)	48 (24)	9 (11)	430 (28)
Alcohol consumption^b^ (%)						
Never	74 (18)	252 (63)	205 (51)	119 (60)	48 (58)	698 (47)
Social	169 (40)	86 (22)	67 (17)	49 (25)	5 (6)	376 (25)
Regular	120 (29)	60 (15)	82 (21)	18 (9)	30 (36)	310 (21)
Heavy	20 (5)	2 (1)	46 (12)	0 (0)	0 (0)	68 (5)
**Educational and psychosocial characteristics**						
Educational level^c^ (%)						
None	7 (2)	0 (0)	35 (9)	14 (7)	28 (34)	84 (6)
Primary school	65 (16)	95 (24)	178 (45)	30 (15)	46 (55)	414 (28)
Middle school	249 (59)	91 (23)	6 (2)	51 (26)	0 (0)	397 (26)
High School	3 (1)	17 (5)	164 (41)	44 (22)	2 (2)	230 (15)
Intermediate or post-high school diploma	20 (5)	52 (13)	17 (4)	60 (30)	7 (8)	156 (10)
Graduate or post-graduate	1 (0)	11 (3)	1 (0)	6 (3)	0 (0)	19 (1)
TB health literacy score^d^ [median (IQR)]	7 (5–9)	5 (4–7)	7 (4–9)	7 (5–9)	2 (1–4)	6 (4–8)
**Economic characteristics**						
Unemployed or retired^f^ (%)	280 (67)	257 (64)	144 (36)	88 (44)	55 (66)	824 (55)
Personal monthly income^g^ (%)						
Tier 1	271 (65)	222 (56)	87 (22)	22 (11)	37 (45)	639 (43)
Tier 2	124 (30)	104 (26)	125 (31)	102 (51)	18 (22)	473 (32)
Tier 3	16 (4)	44 (11)	25 (6)	23 (12)	1 (1)	109 (7)
Tier 4	3 (<1)	3 (<1)	0 (0)	10 (5)	5 (6)	21 (1)
Household monthly income^g^ (%)						
Tier 1	209 (50)	123 (31)	87 (22)	22 (11)	38 (46)	479 (32)
Tier 2	170 (41)	165 (41)	121 (30)	98 (49)	32 (39)	586 (39)
Tier 3	28 (7)	80 (20)	26 (7)	26 (13)	1 (1)	161 (11)
Tier 4	5 (1)	31 (8)	0 (0)	11 (6)	5 (6)	52 (4)

Abbreviation: IQR, interquartile range

^a^18 patients (1 %) were of unknown HIV status

^b^50 patients (3 %) had an unknown level of alcohol consumption

^c^18 patients (%) had missing level of education data

^d^26 patients (2 %) did not have sufficient data to compute a TB health literacy score

^e^7 (<1 %) of patients did not have sufficient data to compute a Kessler K-10 score

^f^4 patients (<1 %) were missing information about their employment status

^g^The monthly income tiers correspond to Under 600 ZAR, 600–3000 ZAR, 3001–7000 ZAR, and More than 7000 ZAR for the South African sites, and Under 100 US$, 100–300 US$, 301–700 US$, and More than 701 US$ for the other sites, respectively. 22 (2 %) patients were missing data about their personal income, and 7 (1 %) were missing data about household income

### Psychological distress

**Fig. 2:**
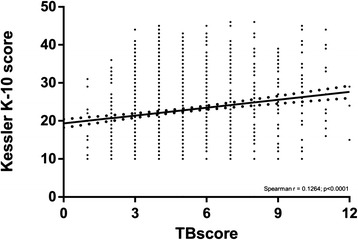
Correlation between psychological distress (Kessler K-10 score) and increased morbidity, measured using a TB-symptom score (TBscore)

Twenty two percent of patients with symptoms of pulmonary TB (335/1502) had a severe level of psychological distress (K-10 score ≥30 [32]) and this was higher in Harare (50 %) and Mbeya (51 %), compared to Lusaka (12 %), Cape Town (8 %) and Durban (8 %). K-10 score strongly positively correlated with TBscore (Spearnman’s Rho 0.1264, *p* <0.0001; Fig. 2). Women had a higher level of psychological distress than men [median (IQR) K-10 score of 24 (18–30) vs. 21 (15–27); *p* <0.0001]. HIV-infected patients had a higher level of psychological distress compared to those who were HIV-uninfected [24 (17–30) vs. 20 (14.25-25.0); *p* <0.0001]. In a multivariate linear regression model for psychological distress (Table 2), female gender [estimate (95 % CI) = 1.47 (2.28, 0.62); *p* = 0.0007], previous TB [estimate = 0.98 (0.09-1.87); *p* = 0.0304], increased TBscore [estimate = 1 (0.80, 1.20); *p* <0.0001], and heavy alcohol usage [estimate = 3.08 (1.26, 4.91); *p* = 0.0010], were associated with increased K-10 score. Culture-confirmed TB was not associated with increased K-10 score [23 (16, 29) vs. 22 (15–29) in culture-negative patients; *p* = 0.2727]. In a multivariate logistic regression model for severe psychological distress (K-10 ≥ 30), TBscore [OR (95 % CI) = 1.30 (1.20, 1.41); *p* <0.0001] and site [7.96 (3.61, 18.26) for Mbeya (*p* <0.0001), and 15.77 (8.81, 30.17) for Harare (*p* <0.0001)] were the only independent predictors.

**Table 2:** Unadjusted and adjusted baseline associates of psychological distress (K-10 score) at recruitment

	Univariate analysis		Multivariate analysis	
	Crude estimate (95 % CI)	*P*-value	Adjusted estimate (95 % CI)	*P*-value
**Demographic and clinical characteristics**				
Age	0.045 (0.010. 0.083)	0.0132	0.031 (−0.003, 0.064)	0.0702
Male	−2.499 (−3.371, −1.627)	<0.0001	−1.447 (−2.278, −0.617)	0.0007
Previously had TB	−0.541 (−1.561, 0.479)	0.2987	0.980 (0.094, 1.866)	0.0304
HIV-infected	3.059 (2.172, 3.946)	<0.0001	0.061 (−0.763, 0.885)	0.8844
TBscore	0.698 (0.481, 0.914)	<0.0001	1.000 (0.802, 1.199)	<0.0001
Allocation arm Xpert MTB/RIF	−0.245 (−1.118, 0.628)	0.5824	-	-
Culture-confirmed TB	−0.450 (−1.468, 0.568)	0.3866	-	-
**Substance use**				
Tobacco smoker	−3.123 (−4.078, −2.169)	<0.0001	-	-
Alcohol consumption				
Never	1.00 (reference)	N/A	1.00 (reference)	N/A
Social	−3.124 (−4.203, −2.045)	<0.0001	−0.599 (−1.592, 0.395)	0.2378
Regular	−2.061 (−3.212, −0.910)	0.0005	−0.001 (−1.041, 1.039)	0.9991
Heavy	−1.362 (−3.496, 0.773)	0.2115	3.082 (1.256,4.907)	0.0010
**Educational characteristics**				
Educational level				
None	−6.496 (−10.722, −2.251)	0.0027	−1.667 (−5.155, 1.82)	0.3489
Primary school	−3.751 (−7.664, 0.160)	0.0604	0.420 (−2.771, 3.610)	0.7966
Middle school	−5.224 (−9.140, −1.308)	0.0090	−0.528 (−3.762, 2.707)	0.7492
High School	−4.792 (−8.700, −0.884)	0.0164	−0.324 (−3.508, 2.860)	0.8418
Intermediate or post-high school diploma	−3.5906 (−7.674, 0.493)	0.0850	0.988 (−2.378, 4.354)	0.5651
Graduate or post-graduate	0.00 (reference)	N/A	0.00 (reference)	N/A
TB health literacy score	−0.409 (−0.551, −0.267)	<0.0001	-	-
**Economic characteristics**				
Unemployed or retired	2.877 (2.012, 3.742)	<0.0001	-	-
Personal monthly income				
Tier 1	1.578 (−1.982, 5.139)	0.3850	-	-
Tier 2	−1.291 (−4.879, 2.296)	0.4806	-	-
Tier 3	−0.804 (−4.637, 3.028)	0.6809	-	-
Tier 4	0.00 (reference)	N/A	-	-
Household monthly income				
Tier 1	−1.802 (−4.187, 0.583)	0.1389	-	-
Tier 2	−2.352 (−4.718, 0.015)	0.0516	-	-
Tier 3	−2.066 (−4.676, 0.543)	0.1209	-	-
Tier 4	0.00 (reference)	N/A	-	-
**Sites**				
Cape Town	0.565 (−0.637, 1.767)	0.3569	1.525 (0.142, 2.907)	0.0309
Harare	10.648 (9.440, 11.856)	<0.0001	11.070 (9.802, 12.340)	<0.0001
Lusaka	−0.636 (−1.845, 0.574)	0.3031	−1.281 (−2.623, 0.061)	0.0616
Mbeya	8.423 (6.603, 10.243)	<0.0001	6.923 (4.9945, 8.911)	<0.0001
Durban	0.00 (reference)	N/A	0.00 (reference)	N/A

Cells marked with a dash indicate variables excluded from the final multivariate model

### Anti-TB treatment adherence

Twenty six percent (69/261) of patients with confirmed TB who were placed on treatment and followed-up at two or six months were non-adherent. These patients had a higher K-10 score [median (IQR) 27.0 (23–33) vs. 21.5 (16–29); *p* <0.0001)] and a lower level of TB-related health literacy [5.5 (4–7) vs. 7 (4–8); *p* = 0.0131)] at recruitment than those who were adherent. When multivariable adjustments were performed to account for site-, baseline morbidity-, and other clinical- and socioeconomic differences (Table 3), the significant association of K-10 score [OR = 1.082 (1.033, 1.137); *p* = 0.0012] and health literacy [OR = 0.798 (0.696, 0.906); *p* = 0.0008] with non-adherence persisted. TB-related morbidity at recruitment [OR = 0.639 (0.497, 0.797); *p* = 0.0002] and heavy alcohol usage [OR = 14.83 (2.083, 122.9); *p* = 0.0002] were also associated with an elevated risk of treatment non-adherence. When psychological distress was included in the logistic regression model as a dichotomous variable, a K-10 score ≥30 was associated with a 2.29-fold (1.033, 5.126; *p* = 0.0417) relative increase in the relative risk of non-adherence (provided the other variables held constant).

**Table 3:** Unadjusted and adjusted baseline associates of treatment non-adherence in patients with culture-confirmed TB

	Univariate analysis		Multivariate analysis	
	Crude odds ratio (95 % CI)	*P*-value	Adjusted odds ratio (95 % CI)	*P*-value
**Demographic and clinical characteristics**				
Age	0.997 (0.970, 1.024)	0.8457	0.988 (0.947, 1.029)	0.5563
Male	0.859 (0.491, 1.517)	0.5971	0.895 (0.402, 2.246)	0.8951
Previously had TB	0.729 (0.346, 1.445)	0.3832	-	-
HIV-infected	1.227 (0.693, 2.215)	0.4889	1.358 (0.557, 3.421)	0.5054
TBscore	0.868 (0.748, 0.998)	0.0524	0.639 (0.497, 0.797)	0.0001
Allocation arm Xpert MTB/RIF	2.038 (1.161, 3.648)	0.0144	2.174 (0.985, 5.015)	0.05993
K-10 score	1.073 (1.039, 1.112)	<0.0001	1.082 (1.033, 1.137)	0.0012
**Substance use**				
Tobacco smoker	0.651 (0.325, 1.239)	0.2055	-	-
Alcohol consumption				
Never	1.00 (reference)	N/A	1.00 (reference)	N/A
Social	0.629 (0.296, 1.279)	0.2122	0.505 (0.177, 1.364)	0.1874
Regular	0.701 (0.327, 1.435)	0.3422	0.707 (0.243, 1.986)	0.5137
Heavy	2.371 (0.624, 9.029)	0.1933	14.83 (2.083, 122.9)	0.0090
**Educational characteristics**				
Educational level				
None	0.375 (0.030, 4.299)	0.4167	-	-
Primary school	0.500 (0.077, 4.022)	0.4668	-	-
Middle school	0.330 (0.049, 2.727)	0.2539	-	-
High School	0.684 (0.107, 5.428)	0.6874	-	-
Intermediate or post-high school diploma	0.789 (0.112, 6.743)	0.81183	-	-
Graduate or post-graduate	0.00 (reference)	N/A	-	-
TB health literacy score	0.885 (0.809, 0.967)	0.0071	0.798 (0.696, 0.907)	0.0008
**Economic characteristics**				
Unemployed or retired			-	-
Personal monthly income				
Tier 1	*	0.9839	-	-
Tier 2	*	0.9840	-	-
Tier 3	*	0.9838	-	-
Tier 4	1.00 (reference)	N/A	-	-
Household monthly income				
Tier 1	0.353 (0.102, 1.181)	0.089747	0.288 (0.064, 1.264)	0.0997
Tier 2	0.275 (0.082, 0.898)	0.031567	0.350 (0.082, 1.445)	0.1462
Tier 3	0.202 (0.039, 0.900)	0.041743	0.156 (0.024, 0.922)	0.0462
Tier 4	1.00 (reference)	N/A	1.00 (reference)	N/A
**Sites**				
Cape Town	0.427 (0.121 1.593)	0.1868	-	-
Harare	3.709 (1.308, 12.285)	0.01956	-	-
Lusaka	1.036 (0.358, 3.461)	0.9499	-	-
Mbeya	0.900 (0.193, 3.980)	0.8888	-	-
Durban	1.00 (reference)	N/A	-	-

Cells marked with a dash indicate variables excluded from the final multivariate model

*indicates where accurate estimation of the odds ratio failed due to too few observations

### Morbidity

Patients placed on treatment who had a ≥25 % improvement in their morbidity score after six months had a higher level of psychological distress at baseline than those who did not [median (IQR) TBscore of 23 (17–28) vs. 17 (10–23); *p* <0.0001]. When correlates of per-patient changes in morbidity between baseline and six months were examined in a multivariate analysis (Table 4), patients who were younger [estimate (95 % CI) = −0.01 (−0.02, −0.01); *p* = 0.0096], female [estimate = 0.26 (0.04, 0.49); *p* = 0.0208], had previous TB [estimate = −0.01 (−0.02, −0.01); *p* = 0.0096], were HIV-infected [estimate = 0.41 (0.16, 0.65); *p* = 0.0011], were culture-positive [estimate = 0.59 (0.32, 0.85); *p* <0.0001] or who had a higher K-10 score at recruitment [estimate = 0.41 (0.02, 0.06); *p* <0.0001] had the largest improvement in their morbidity.

**Table 4:** Unadjusted and adjusted baseline associates of improvement in TB symptom score (TBscore) in patients started on anti-TB treatment and followed-up at six months

	Univariate analysis		Multivariate analysis	
	Crude estimate (95 % CI)	*P*-value	Adjusted estimate (95 % CI)	*P*-value
**Demographic and clinical characteristics**				
Age	0.002 (−0.007, 0.010)	0.7101	−0.013 (−0.022, −0.003)	0.0097
Male	0.357 (0.156, 0.557)	0.0005	0.263 (0.040, 0.486)	0.0208
Previously had TB	0.349 (0.124, 0.574)	0.0024	−0.228 (−0.491, 0.0353)	0.0901
HIV-infected	−0.365 (−0.568, −0.162)	0.0005	0.406 (0.163, 0.6493)	0.0011
TBscore	0.212 (0.157, 0.266)	<0.0001		
Allocation arm Xpert MTB/RIF	0.059 (−0.142, 0.261)	0.5607	-	-
Culture-confirmed TB	0.368 (0.128, 0.607)	0.0027	0.587 (0.323, 0.850)	<0.0001
K-10 score	−0.045 (−0.056, −0.031)	<0.0001	0.041 (0.024, 0.057)	<0.0001
**Substance use**				
Tobacco smoker	0.340 (0.127, 0.554)	0.0018	-	-
Alcohol consumption				
Never	0.00 (reference)	N/A	-	-
Social	0.088 (−0.151, 0.327)	0.4722	-	-
Regular	0.264 (0.000, 0.527)	0.0501	-	-
Heavy	1.346 (0.816, 1.876)	<0.0001	-	-
**Educational characteristics**				
Educational level				
None	1.711 (0.742, 2.679)	0.0006	-	-
Primary school	1.075 (0.216, 1.933)	0.0143	-	-
Middle school	0.703 (−0.147, 1.552)	0.1052	-	-
High School	0.718 (−0.137, 1.572)	0.1002	-	-
Intermediate or post-high school diploma	0.404 (−0.492, 1.299)	0.3771	-	-
Graduate or post-graduate	0.00 (reference)	N/A		
TB health literacy score	0.0450 (0.011, 0.079)	0.0102	-	-
**Economic characteristics**				
Unemployed or retired	−0.087 (−0.293, 0.120)	0.4105	-	-
Personal monthly income				
Tier 1	0.271 (−0.578, 1.119)	0.53181	-	-
Tier 2	0.354 (−0.503, 1.211)	0.4180	-	-
Tier 3	−0.066 (−0.977, 0.846)	0.8879	-	-
Tier 4	0.00 (reference)	N/A	-	-
Household monthly income				
Tier 1	0.948 (0.427, 1.469)	0.0004	-	-
Tier 2	0.749 (0.230, 1.269)	0.0048	-	-
Tier 3	0.282 (−0.292, 0.855)	0.3360	-	-
Tier 4	0.00 (reference)	N/A	-	-
**Sites**			-	-
Cape Town	0.322 (0.060, 0.585)	0.01635	−1.306 (−1.691, −0.921)	<0.0001
Harare	−0.926 (−1.198, −0.655)	<0.0001	−0.471 (−0.883, −0.059)	0.0253
Lusaka	2.415 (2.091, 2.740)	<0.0001	−2.393 (−2.822, −1.965)	<0.0001
Mbeya	0.5904 (0.202, 0.979)	0.0030	0.367 (−0.226, 0.960)	0.2259
Durban	0.00 (reference)	N/A	0.00 (reference)	N/A

### Mortality

Patients who started treatment and died during the six month follow-up period had a higher level of psychological distress at recruitment compared to those that were alive at six months [median (IQR) K-10 scores of 26.5 (20.25-33) vs. 24 (17–30); *p* = 0.0268]. Morbidity at recruitment was, however, the strongest predictor of mortality during the six month follow-up period in a multivariate model (Additional file 1: Table S4) [OR = 1.43 (1.28, 1.69); *p* <0.0001]. Older age [OR = 1.03 (1.01, 1.05); *p* = 0.0013], HIV-infection [OR = 3.12 (1.89, 5.55); *p* <0.0001], and unemployment [OR = 1.79 (1.11, 2.93); *p* = 0.0180] were also significant determinants of death, whereas psychological distress was not [OR = 1.028 (0.99, 1.07); *p* = 0.1578 in the initial model).

### TB health literacy

Patients with culture-confirmed TB had, at diagnosis, a similar TB-related health literacy score than those without TB [median (IQR) 7 (4–8) vs. 6 (4–8); *p* = 0.7880]. In a multivariate analysis (Additional file 1: Table S5), people who had a lower education level [estimate (95 % CI) of −4.30 (−5.81, −2.78) and *p* <0.0001 for those who had no formal education] and said they consumed alcohol socially [estimate = −0.54 (−0.97, −0.10); *p* = 0.0159] had a lower literacy score. Previous TB was associated with increased TB health literacy [estimate = 0.43 (0.04, 0.82); *p* = 0.0301] in the model.

### Cough duration

Patients with TB who reported themselves to have been coughing for at least two weeks prior to presenting to the clinic (*n* = 1402) (and are hence likely to have transmitted more disease than those who had not) experienced a delay in seeking care. These patients had higher psychological distress than those who had not had a cough for at least two weeks (*n* = 100) [K-10 score of 22 (15–29) vs. 13 (11.75-17.25); *p* = 0.0002)]. However, in a multivariable model for cough duration restricted to patients in the former group, HIV-infection [estimate = −14.85 (−27.87, −1.838); *p* = 0.0267], and heavy alcohol usage [estimate = 85.14 (44.77, 125.5); *p* <0.0001] were the only significant associates of cough duration (Additional file 1: Table S6).

### Employment

In Mbeya and Durban, the proportion of patients who were unemployed was greater amongst those with culture-confirmed TB compared to those with without TB [69 % vs. 37 % for Mbeya (*p* = 0.0059); 66 % vs. 39 % for Durban (*p* = 0.0045)], however, this proportion did not differ significantly at the other sites. Amongst patients who were unemployed, 23 % (184/797) said their unemployment was due to their current illness. When psychological distress at recruitment was compared between patients who later reported at follow-up that their illness had, since diagnosis, affected their employment and those who had reported it had not, baseline K-10 scores were elevated amongst the former group [22 (16–30) vs. 19 (14–24); *p* = 0.0013], however, when multivariable adjustments were performed (Additional file 1: Table S7), K-10 score was not an independent predictor of this outcome [OR = 1.02 (0.978-1.064); *p* = 0.3465].

## Discussion

This large multicentre study examined the relationship of psychological distress, alcohol use, health literacy, clinical morbidity, and socioeconomic factors, with treatment non-adherence and clinical outcomes. Our key findings were that: (i) heavy alcohol usage, female gender, increased morbidity, and previous TB are associated with increased levels of psychological distress amongst patients with symptoms of TB, however, TB status is not; (ii) increased psychological distress, heavy alcohol usage, decreased health literacy and decreased morbidity are independently associated with non-adherence to treatment; (iii) patients who were more psychologically distressed at treatment initiation had the greatest clinical improvement in symptoms, provided they were adherent; and (iv) HIV-infection and heavy alcohol usage are associated with a delay in seeking care (defined as duration of coughing before presentation) amongst patients with culture-confirmed TB.

We found that for every point increase in K-10 score, there was an 8 % increase in the odds of treatment non-adherence over a six month period, after adjustment for site-specific interactions (provided other factors remained equal). Furthermore, for each one unit increase in TB health literacy score, there was a 20 % reduction in the relative risk of treatment non-adherence. This suggests that education about TB at the time of treatment initiation is presently inadequate, and that this results in non-adherence. Although rates of psychological distress have previously been surveyed in patients seeking care for TB [14, 33] and HIV [34], our study is, to the best of our knowledge, the first to demonstrate a link between psychological distress and anti-TB treatment non-adherence, and the first to show that poor level of TB-related health literacy, which we show is itself associated with a low level of formal education, is a risk factor for non-adherence. A study from Peru [19] has previously demonstrated an association between major depressive episodes and cocaine use with a composite outcome comprised of anti-TB treatment abandonment, however, this study did not examine non-adherence specifically.

Our study showed approximately half of patients presenting to our primary care TB clinics in Harare and Mbeya to have a severe level (K-10 ≥ 30) of psychological distress, similar to that previous reported amongst Ethiopian patients infected with HIV or TB or both [33]. These high levels of psychological distress likely resulted from the high local prevalence of risk factors. For example, Harare had the highest rate of HIV-infection out of the five sites, whereas more patients in Mbeya reported themselves to consume alcohol regularly than at any other site. In South Africa, the rate of severe psychological distress detected is identical (8 %) to that reported in an earlier nationally-representative survey of mental illness [32]. To the best of our knowledge, our study is the first to report the use of the K-10 questionnaire in Southern African countries besides South Africa.

We found women and patients living with HIV to have a significantly higher level of psychological distress than men or HIV-uninfected patients, respectively. According to stratifications of the K-10 score previously performed in South Africa (where a score of 20–24 was graded as moderate) [32], these differences, although significant, would not constitute an overall increase in risk classification level. We found heavy alcohol use to be associated with increased psychological distress and, as demonstrated by others [9, 35, 36], to be associated with non-adherence. Heavy alcohol users are more likely to experience social marginalisation and have side effects from their anti-TB medication [5], which may further worsen non-adherence. These data suggest that screening for common mental disorders and alcohol abuse should, together with measures to educate patients about TB, be strengthened. Education and counselling to promote adherence can be effective [37] and our data suggest they should be targeted at high risk individuals who drink alcohol heavily, have severe psychological distress, and have low TB health literacy. Of note in this study, is the strong, bidirectional linkage between clinical signs and symptoms and psychological distress.

We found HIV-infection and heavy alcohol usage to be associated with a longer duration of cough before in presentation to the clinic, which is suggestive that these patients may be responsible for more transmission than those who present earlier. This is likely exacerbated by the tendency of patients who are heavy users of alcohol to congregate in settings permissive for transmission [5]. Both alcohol use and HIV have previously been described to be associated with a delay in seeking care [38, 39], and our study reiterates the need for active case finding targeted at these individuals.

Our study has limitations. Firstly, we reviewed DOTS treatment cards, and thus patients who visited the clinic but did not take their medication may have been missed. This method is, however, widely used for research [40–42], as it is practical and cost effective, especially compared to methods that require monitoring drug concentrations. We also only recorded whether patients were compliant, but did not capture data about the proportion of doses taken. Consequently, we were unable to discriminate between patients who only missed a few doses and those who were completely non-compliant and had defaulted (which is defined as no treatment for a continuous period of two months by the World Health Organisation [43]). Secondly, we measured psychological distress once-off on a cross-sectional basis, when ideally it should be measured longitudinally over the course of treatment, however, we wanted to know if interventions at diagnosis (which is when the longest patient-health system encounter occurs) could be effective. Thirdly, the parent study was a randomised controlled trial of a diagnostic test, however, in none of our multivariable analyses was allocation arm a significant associate of model outcome. Fourthly, we did not use a standard measure of alcohol consumption severity, such as AUDIT [44]. Finally, our study was not an interventional study to measure the impact of counselling for reducing non-adherence.

## Conclusions

Overall, our study found severe psychological distress to be frequent amongst patients seeking care for TB in Southern Africa. We found a clear linkage between psychological distress, alcohol use, health literacy, and clinical morbidity with non-adherence to anti-TB treatment, which was independent of socioeconomic factors and site-specific interactions. Psychological distress was strongly co-associated with clinical signs and symptoms. Screening for psychological distress could, together with counselling to reduce alcohol consumption and improve patients’ knowledge about TB, reduce treatment non-adherence.

## Additional file

Additional file 1:
**Information about the screening questionaires used, and supplementary multivariate analysis tables.**
