# Supplementary material for: Psychological distress and its relationship with non-adherence to TB treatment: a multicentre study
Source: BMC Infect Dis. 2015 Jul 1;15:253. doi: 10.1186/s12879-015-0964-2 (PMC4487582; doi:10.1186/s12879-015-0964-2)
Supplement: Additional file 1: — Information about the screening questionaires used, and supplementary multivariate analysis tables. [file 12879_2015_964_MOESM1_ESM.doc]

**Supplementary information**

**Table S1.** Nurse-administered K10-standardised mental illness screening questionnaire used in the study.

| **K10-Standardised Mental illness Screening Questionnaire** | | | | | |
| --- | --- | --- | --- | --- | --- |
| The following questions ask about how you have been feeling during the past four weeks. For each question, please circle the number that best describes how often you had this feeling | | | | | |
| **In the past 4 weeks** | None of  the time | A little of  the time | Some of  the time | Most of  the time | All of  the time |
| 1.1. About how often did you feel tired out for no good reason? | 1 | 2 | 3 | 4 | 5 |
| 1.2. About how often did you feel nervous? | 1 | 2 | 3 | 4 | 5 |
| 1.3. About how often did you feel so nervous that nothing could calm you down? | 1 | 2 | 3 | 4 | 5 |
| 1.4. About how often did you feel hopeless? | 1 | 2 | 3 | 4 | 5 |
| 1.5. About how often did you feel restless or fidgety? | 1 | 2 | 3 | 4 | 5 |
| 1.6. About how often did you feel so restless you could not sit still? | 1 | 2 | 3 | 4 | 5 |
| 1.7. About how often did you feel depressed? | 1 | 2 | 3 | 4 | 5 |
| 1.8. About how often did you feel that everything is an effort? | 1 | 2 | 3 | 4 | 5 |
| 1.9 About how often did you feel so sad that nothing could cheer you up? | 1 | 2 | 3 | 4 | 5 |
| 1.10. About how often did you feel worthless? | 1 | 2 | 3 | 4 | 5 |

Adapted from Kessler *et al.* (2002), and Kessler *et al.* (2003)

**Table S2.** TB health literacy questionnaire

| QUESTIONS | TRUE | FALSE | NOT SURE |
| --- | --- | --- | --- |
| 10.1. TB can be spread by coughing, sneezing or spitting |  |  |  |
| 10.2. If you live or work with someone who has TB, you can get TB. |  |  |  |
| 10.3. If you are exposed once to someone with TB you can still get it |  |  |  |
| 10.4. People who are homeless or who live on the street are more likely to get TB |  |  |  |
| 10.5. Some types of people, like migrants or foreigners from different countries (like Somalis or Zimbabwe) are more likely to get TB |  |  |  |
| 10.6. If your immune system or the way your body fights disease is not working properly, it is easier to get TB |  |  |  |
| 10.7. If you have HIV/AIDS, it is easier to get TB |  |  |  |
| 10.8. TB is hard to treat |  |  |  |
| 10.9. People can become resistant to the medication used to treat TB and the medication can stop working |  |  |  |
| 10.10. TB disease can severely damage a person’s lungs |  |  |  |
| 10.11. You can tell if someone you know has TB disease |  |  |  |
| 10.12. TB is caused by a germ or bug |  |  |  |
| 10.13. People can die from TB if it is not treated |  |  |  |

A point is assigned for every correct answer. No points are assigned for a “not sure” answer. A point is deducted for every incorrect answer.

**Table S3.** Variables used to calculate the TB score as defined by Wejse *et al.* (2008). Each patient was scored at baseline, 2 months and 6 months.

| **Parameters** | **Points assigned** (Maximum score is 13) |
| --- | --- |
| Self-reported | |
| Cough | 1 |
| Haemoptysis | 1 |
| Dyspnoea | 1 |
| Chest pain | 1 |
| Night sweats | 1 |
| Clinically examined | |
| Anaemic conjunctivae | 1 |
| Tachycardia | 1 |
| Positive finding at lung auscultation | 1 |
| Axillary temperature > 37.0 °C | 1 |
| BMI < 18.0 | 1 |
| BMI < 16.0 | 1 |
| MUAC < 220 mm | 1 |
| MUAC < 200 mm | 1 |

Abbreviations: BMI, body mass index; MUAC, middle upper arm circumference.

**Table S4.** Unadjusted and adjusted baseline associates of mortality in patients started on anti-TB treatment.

|  | **Univariate analysis** | | **Multivariate analysis** | |
| --- | --- | --- | --- | --- |
|  | **Crude odds ratio**  (95% CI) | **P-value** | **Adjusted odds ratio**  (95% CI) | **P-value** |
| **Demographic and clinical characteristics** | | | | |
| Age | 1.02 (1.00, 1.03) | 0.0218 | **1.031 (1.012, 1.051)** | **0.0014** |
| Male | 1.346 (0.919, 1.922) | 0.1305 | 1.348 (0.851, 2.153) | 0.2064 |
| Previously had TB | 0.7474 (0.462, 1.167) | 0.2156 | - | - |
| HIV-infected | 2.745 (1.774, 4.389) | <0.0001 | - | - |
| TBscore | 1.40 (1.286, 1.532) | <0.0001 | **1.425 (1.281, 1.587)** | **<0.0001** |
| Allocation arm Xpert MTB/RIF | 0.910 (0.624, 1.326) | 0.6247 | - | - |
| Culture-confirmed TB | 1.211 (0.777, 1.846) | 0.3842 | - | - |
| K-10 score | 1.056 (1.035, 1.082) | <0.0001 | - | - |
| **Substance use** | | | | |
| Tobacco smoker | 0.423 (0.247, 0.685) | 0.0009 | - | - |
| Alcohol consumption |  |  |  |  |
| Never | 1.00 (reference) | N/A | - | - |
| Social | 0.806 (0.503, 1.266) | 0.3574 | - | - |
| Regular | 0.8347 (0.499, 1.355) | 0.4762 | - | - |
| Heavy | 0.6150 (0.146, 1.765) | 0.4291 | - | - |
| **Educational characteristics** | | | | |
| Educational level |  |  |  |  |
| None | 0.952 (0.249, 4.672) | 0.9463 | - | - |
| Primary school | 0.778 (0.242, 3.472) | 0.7020 | - | - |
| Middle school | 0.234 (0.068, 1.083) | 0.0335 | - | - |
| High School | 0.566 (0.174, 2.537) | 0.3882 | - | - |
| Intermediate or post-high school diploma | 0.438 (0.114, 2.145) | 0.2545 | - | - |
| Graduate or post-graduate | 1.00 (reference) | N/A |  |  |
| TB health literacy score | 0.927 (0.873, 0.985) | 0.0134 | - | - |
| **Economic characteristics** | | | | |
| Unemployed or retired | 1.287 (0.876, 1.913) | 0.2046 | **1.793 (1.110, 2.929)** | **0.0180** |
| Personal monthly income |  |  |  |  |
| Tier 1 | * | 0.9824 | - | - |
| Tier 2 | * | 0.9821 | - | - |
| Tier 3 | * | 0.9816 | - | - |
| Tier 4 | 1.00 (reference) | N/A | - | - |
| Household monthly income |  |  |  |  |
| Tier 1 | 0.659 (0.241, 2.319) | 0.4586 | 0.473 (0.162, 1.74) | 0.2068 |
| Tier 2 | 1.196 (0.458, 4.1) | 0.7429 | 1.137 (0.418, 3.998) | 0.8190 |
| Tier 3 | 1.425 (0.495, 5.16) | 0.5437 | 1.593 (0.532, 5.931) | 0.4382 |
| Tier 4 | 1.00 (reference) | N/A | 1.00 (reference) | N/A |
| **Sites** |  |  |  |  |
| Cape Town | 0.209 (0.089, 0.466) | 0.0002 | - | - |
| Harare | 1.224 (0.688, 2.286) | 0.5074 | - | - |
| Lusaka | 1.159 (0.606, 2.282) | 0.6607 | - | - |
| Mbeya | 2.118 (0.987, 4.554) | 0.0525 | - | - |
| Durban | 1.00 (reference) | N/A | - | - |

Cells marked with a dash indicate variables excluded from the final multivariate model. * indicates where accurate estimation of the odds ratio failed due to too few observations.

**Table S5.** Unadjusted and adjusted baseline associates of health literacy.

|  | **Univariate analysis** | | **Multivariate analysis** | |
| --- | --- | --- | --- | --- |
|  | **Crude estimate**  (95% CI) | **P-value** | **Adjusted estimate**  (95% CI) | **P-value** |
| **Demographic and clinical characteristics** | | | | |
| Age | -0.008 (-0.021, 0.005) | 0.2264 | 0.011 (-0.004, 0.026) | 0.1650 |
| Male | 0.340 (0.026, 0.655) | 0.0338 | 0.1103 (-0.263, 0.484) | 0.5627 |
| Previously had TB | 0.926 (0.566, 1.286) | <0.0001 | **0.431 (0.042, 0.820)** | **0.0301** |
| HIV-infected | -0.357 (-0.677, -0.038) | 0.0286 | -0.143 (-0.510, 0.225) | 0.4464 |
| TBscore | -0.093 (-0.171, -0.015) | 0.0193 | **-** | **-** |
| Allocation arm Xpert MTB/RIF | -0.105 (-0.417, 0.206) | 0.5070 | - | - |
| Culture-confirmed TB | -0.025 (-0.387. 0.338) | 0.8945 | - | - |
| K-10 score | -0.052 (-0.070, -0.034) | <0.0001 | - | - |
| **Substance use** | | | | |
| Tobacco smoker | 0.7510 (0.409, 1.093) | <0.0001 | - | - |
| Alcohol consumption |  |  |  |  |
| Never | 1.00 (reference) | N/A | 1.00 (reference) | N/A |
| Social | 0.197 (-0.161, 0.611) | 0.2526 | **-0.536 (-0.979, -0.101)** | **0.0159** |
| Regular | 0.210 (-0.322, 0.502) | 0.6690 | -0.279 (-0.732, 0.174) | 0.2277 |
| Heavy | 0.391 (0.030, 1.562) | 0.0419 | 0.280 (-0.540, 1.100) | 0.5029 |
| **Educational characteristics** | | | | |
| Educational level |  |  |  |  |
| None | -3.215 (-4.693, -1.737) | <0.0001 | **-4.296 (-2.782, 0.772)** | **<0.0001** |
| Primary school | -1.761 (3.124, -0.399) | 0.0114 | **-2.622 (-3.943, -1.300)** | **0.0001** |
| Middle school | -0.688 (-2.051, 0.676) | 0.3231 | **-1.947 (-3.286, -0.608)** | **0.0044** |
| High School | -0.777 (-2.139, 0.584) | 0.2632 | -1.786 (-3.100, -0.472) | 0.0078 |
| Intermediate or post-high school diploma | 0.073 (-1.350, 1.495) | 0.9204 | -0.986 (-2.373, 0.402) | 0.1640 |
| Graduate or post-graduate | 1.00 (reference) | N/A | 1.00 (reference) | N/A |
| **Economic characteristics** | | | | |
| Unemployed or retired | -0.566 (-0.877, -0.254) | 0.0004 | - | - |
| Personal monthly income |  |  |  |  |
| Tier 1 | -1.498 (-2.813, -0.184) | 0.0257 | - | - |
| Tier 2 | -0.744 (-2.069, 0.580) | 0.2710 | - | - |
| Tier 3 | -0.640 (-2.055, 0.775) | 0.3755 | - | - |
| Tier 4 | 0.00 (reference) | N/A | - | - |
| Household monthly income |  |  |  |  |
| Tier 1 | -1.024 (-1.892, -0.157) | 0.0208 | -1.149 (-1.996, -0.302) | 0.00797 |
| Tier 2 | -0.431 (-1.292, 0.430) | 0.3265 | -0.8035 (-1.631, 0.024) | 0.0572 |
| Tier 3 | -0.624 (1.573, 0.325) | 0.1975 | -1.035 (-1.931, -0.139) | 0.0238 |
| Tier 4 | 0.00 (reference) | N/A | 0.00 (reference) | N/A |
| **Sites** |  |  |  |  |
| Cape Town | 0.108 (-0.380, 0.597) | 0.6634 | 0.339 (-0.287, 0.964) | 0.2890 |
| Harare | -1.464 (-1.955, -0.973) | <0.0001 | **-1.305 (-1.869, -0.742)** | **<0.0001** |
| Lusaka | -0.561 (-1.055, -0.067) | 0.0261 | 0.186 (-0.467, 0.839) | 0.5764 |
| Mbeya | -3.917 (-4.657, -3.178) | <0.0001 | **-2.711 (-3.560. -1.86)** | **<0.0001** |
| Durban | 0.00 (reference) | N/A | 0.00 (reference) | N/A |

Cells marked with a dash indicate variables excluded from the final multivariate model.

**Table S6.** Unadjusted and adjusted baseline associates of cough duration in patients with culture-confirmed TB with a cough duration equal or greater to two weeks

|  | **Univariate analysis** | | **Multivariate analysis** | |
| --- | --- | --- | --- | --- |
|  | **Crude estimate**  (95% CI) | **P-value** | **Adjusted estimate**  (95% CI) | **P-value** |
| **Demographic and clinical characteristics** | | | | |
| Age | 0.235 (0.051, 0.420) | 0.0124 | 0.4579 (-0.134, 1.050) | 0.1314 |
| Male | 4.682 (-5.354, 14.718) | 0.3616 | -2.069 (-15.98, 11.84) | 0.7711 |
| Previously had TB | -3.366 (-15.898, 9.166) | 0.599 | **-** | **-** |
| HIV-infected | -5.446 (-15.824. 4.931) | 0.3048 | **-14.86, -27.87. -1.838)** | **0.0267** |
| TBscore | 0.747, -0.370, 1.860) | 0.1903 | **-** | **-** |
| Allocation arm Xpert MTB/RIF | -5.987 (-15.812, 3.839) | 0.2337 | - | - |
| Culture-confirmed TB | 3.081 (-2.037, 8.200) | 0.2383 | - | - |
| K-10 score | 0.311 (0.054, 0.567) | 0.0178 | - | - |
| **Substance use** | | | | |
| Tobacco smoker | 0.111 (-10.873, 11.094) | 0.9843 | - | - |
| Alcohol consumption |  |  |  |  |
| Never | 1.00 (reference) | N/A | 1.00 (reference) | N/A |
| Social | -0.616 (-12.600, 11.368) | 0.9198 | -3.318 (19.420, 12.780) | 0.6868 |
| Regular | -4.061 (-16.725, 8.602) | 0.5303 | -6.234 (-22.040, 9.568) | 0.4406 |
| Heavy | 70.139 (37.768, 102.509) | <0.0001 | **85.140 (44.770, 125.000)** | **<0.0001** |
| **Educational characteristics** | | | | |
| Educational level |  |  |  |  |
| None | -18.833 (-54.001, 16.339) | 0.2958 | -27.220 (-70.670, 16.230) | 0.2214 |
| Primary school | 0.773 (-30.068, 31.614) | 0.9609 | -11.580 (-46.704, 23.580) | 0.5194 |
| Middle school | -8.316 (-39.279, 22.648) | 0.5992 | -8.134 (-42.120, 25.860) | 0.6397 |
| High School | 0.219 (-30.718, 31.157) | 0.9889 | -5.006 (-37.740. 27.730) | 0.7648 |
| Intermediate or post-high school diploma | -11.533 (-45.086, 22.019) | 0.5012 | -15.370 (-52.960, 22.220) | 0.4241 |
| Graduate or post-graduate | 1.00 (reference) | N/A | 1.00 (reference) | N/A |
| TB health literacy score | 0.291 (-0.479, 1.069) | 0.4595 | - | - |
| **Economic characteristics** | | | | |
| Unemployed or retired | 1.046 (-8.991, 11.082) | 0.8384 | - | - |
| Personal monthly income |  |  |  |  |
| Tier 1 | 10.094 (-28.001, 48.188) | 0.6042 | 4.558 (-35.680, 44.790) | 0.8246 |
| Tier 2 | 6.000 -32.692, 44.692) | 0.7615 | -11.740 (-53.600, 30.110) | 0.5832 |
| Tier 3 | 32.235 (-9.335, 73.806) | 0.1303 | 17.850 (-26.540, 62.250) | 0.4318 |
| Tier 4 | 0.00 (reference) | N/A | 0.00 (reference) | N/A |
| Household monthly income |  |  |  |  |
| Tier 1 | 1.035 (-32.737, 23.807) | 0.9291 | - | - |
| Tier 2 | -6.323 (-28.690, 16.044) | 0.5802 | - | - |
| Tier 3 | 12.656 (-13.385, 38.696) | 0.3421 | - | - |
| Tier 4 | 0.00 (reference) | N/A | - | - |
| **Sites** |  |  |  |  |
| Cape Town | 5.248 (-11.201. 21.696) | 0.5324 | -2.289 (-25.820, 21.240) | 0.8490 |
| Harare | 10.912 (-4.990, 26.814) | 0.1801 | 6.112 (-15.120, 27.350) | 0.5735 |
| Lusaka | 13.066 (-3.004, 30.137) | 0.1351 | 18.390 (-6.324, 43.110**)** | 0.1467 |
| Mbeya | 0.00 (reference) | N/A | 0.00 (reference) | N/A |
| Durban | * |  | * |  |

Cells marked with a dash indicate variables excluded from the final multivariate model. *Durban had no culture-confirmed TB cases with a cough greater than two weeks and an absolute known cough duration. Mbeya is therefore here used as a reference category for the site variable.

**Table S7.** Unadjusted and adjusted baseline associates of illness impacting employment between diagnosis and six month follow-up amongst patients started on treatment

|  | **Univariate analysis** | | **Multivariate analysis** | |
| --- | --- | --- | --- | --- |
|  | **Crude odds ratio**  (95% CI) | **P-value** | **Adjusted odds ratio**  (95% CI) | **P-value** |
| **Demographic and clinical characteristics** | | | | |
| Age | 1.016 (0.993, 1.039) | 0.1753 | **0.029 (0.003, 0.224)** | **0.0010** |
| Male | 1.174 (0.719, 1.955) | 0.5286 | 1.010 (0.986, 1.040) | 0.3487 |
| Previously had TB | 1.151 (0.672, 1.931) | 0.5997 | 1.260 (0.693, 2.240) | 0.4456 |
| HIV-infected | 1.028 (0.648, 1.644) | 0.9056 | - | - |
| TBscore | 1.094 (0.975, 1.225) | 0.1238 | - | - |
| Allocation arm Xpert MTB/RIF | 1.382 (0.880, 2.178) | 0.1609 | - | - |
| Culture-confirmed TB | 0.8739 (0.530, 1.417) | 0.5901 | - | - |
| K-10 score | 1.044 (1.017, 1.073) | 0.0014 | 1.020 (0.982, 1.050**)** | 0.3397 |
| **Substance use** | | | | |
| Tobacco smoker | 0.937 (0.565, 1.526) | 0.7965 | - | - |
| Alcohol consumption |  |  |  |  |
| Never | 1.00 (reference) | N/A | - | - |
| Social | 1.297 (0.753, 2.233) | 0.3468 | - | - |
| Regular | 0.977 (0.529, 1.775) | 0.9408 | - | - |
| Heavy | 0.442 (0.101, 1.369) | 0.2049 | - | - |
| **Educational characteristics** | | | | |
| Educational level |  |  |  |  |
| None | * | 0.9813 | * | 0.9802 |
| Primary school | 0.8205 (0.218, 3.967) | 0.7824 | 1.830 (0.442, 9.490) | 0.4245 |
| Middle school | 0.9905 (0.263, 4.781) | 0.9893 | 0.831 (0.188, 4.480) | 0.8144 |
| High School | 0.7095 (0.191, 3.392) | 0.6281 | 1.390 (0.336, 7.120) | 0.6655 |
| Intermediate or post-high school diploma | 1.8095 (0.457, 9.068) | 0.4225 | 3.260 (0.723, 18.0) | 0.1389 |
| Graduate or post-graduate | 1.00 (reference) | N/A | 1.00 (reference) | N/A |
| TB health literacy score | 1.037 (0.960, 1.122) | 0.3610 | - | - |
| **Economic characteristics** | | | | |
| Personal monthly income |  |  |  |  |
| Tier 1 | 0.708 (0.186, 3.447) | 0.6328 | - | - |
| Tier 2 | 0.956 (0.266, 4.477) | 0.9483 | - | - |
| Tier 3 | 1.193 (0.302, 5.953) | 0.8108 | - | - |
| Tier 4 | 1.00 (reference) | N/A |  |  |
| Household monthly income |  |  |  |  |
| Tier 1 | 0.351 (0.125, 1.013) | 0.0472 | - | - |
| Tier 2 | 0.632 (0.252, 1.673) | 0.3351 | - | - |
| Tier 3 | 0.597 (0.214, 1.725) | 0.3277 | - | - |
| Tier 4 | 1.00 (reference) | N/A | - | - |
| **Sites** |  |  |  |  |
| Cape Town | 3.403 (1.331, 10.523) | 0.0180 | **6.150 (2.160. 20.900)** | **0.0015** |
| Harare | 4.614 ((1.732, 14.683) | 0.0043 | **4.860 (1.590, 17.400)** | **0.0086** |
| Lusaka | 0.794 (0.289, 2.557) | 0.6726 | 1.010 (0.337, 3.540) | 0.9811 |
| Mbeya | 2.560 (0.671, 10.213) | 0.1679 | 3.990 (0.873, 19.400) | 0.0761 |
| Durban | 1.00 (reference) | N/A | 1.00 (reference) | N/A |

Cells marked with a dash indicate variables excluded from the final multivariate model. * indicates where accurate estimation of the odds ratio failed due to too few observations.

**References**

1. Kessler RC, Andrews G, Colpe LJ, Hiripi E, Mroczek DK, Normand S-LT, Walters EE, Zaslavsky AM: **Short screening scales to monitor population prevalences and trends in non-specific psychological distress**. *Psychological medicine* 2002, **32**(6):959-976.

2. Kessler RC, Barker PR, Colpe LJ, Epstein JF, Gfroerer JC, Hiripi E, Howes MJ, Normand S-LT, Manderscheid RW, Walters EE: **Screening for serious mental illness in the general population**. *Archives of general psychiatry* 2003, **60**(2):184-189.

3. Morisky DE, Malotte CK, Choi P, Davidson P, Rigler S, Sugland B, Langer M: **A patient education program to improve adherence rates with antituberculosis drug regimens**. *Health Educ Q* 1990, **17**(3):253-267.

4. Wejse C, Gustafson P, Nielsen J, Gomes VF, Aaby P, Andersen PL, Sodemann M: **TBscore: Signs and symptoms from tuberculosis patients in a low-resource setting have predictive value and may be used to assess clinical course**. *Scandinavian journal of infectious diseases* 2008, **40**(2):111-120.
